# Investigating the evolution of apoptosis in malaria parasites: the importance of ecology

**DOI:** 10.1186/1756-3305-3-105

**Published:** 2010-11-16

**Authors:** Laura C Pollitt, Nick Colegrave, Shahid M Khan, Mohammed Sajid, Sarah E Reece

**Affiliations:** 1 Institute of Evolutionary Biology, University of Edinburgh, Edinburgh, School of Biological Sciences, Edinburgh, EH9 3JT, UK; 2 Leiden Malaria Research group, Department of Parasitology, Leiden University Medical Center, The Netherlands; 3 Centre for Immunity, Infection and Evolution, University of Edinburgh, School of Biological Sciences, Edinburgh, EH9 3JT, UK

## Abstract

Apoptosis is a precisely regulated process of cell death which occurs widely in multicellular organisms and is essential for normal development and immune defences. In recent years, interest has grown in the occurrence of apoptosis in unicellular organisms. In particular, as apoptosis has been reported in a wide range of species, including protozoan malaria parasites and trypanosomes, it may provide a novel target for intervention. However, it is important to understand when and why parasites employ an apoptosis strategy before the likely long- and short-term success of such an intervention can be evaluated. The occurrence of apoptosis in unicellular parasites provides a challenge for evolutionary theory to explain as organisms are expected to have evolved to maximise their own proliferation, not death. One possible explanation is that protozoan parasites undergo apoptosis in order to gain a group benefit from controlling their density as this prevents premature vector mortality. However, experimental manipulations to examine the ultimate causes behind apoptosis in parasites are lacking. In this review, we focus on malaria parasites to outline how an evolutionary framework can help make predictions about the ecological circumstances under which apoptosis could evolve. We then highlight the ecological considerations that should be taken into account when designing evolutionary experiments involving markers of cell death, and we call for collaboration between researchers in different fields to identify and develop appropriate markers in reference to parasite ecology and to resolve debates on terminology.

## Introduction

Apoptosis is a controlled process of programmed cell death by which unwanted or damaged cells are eliminated [1,2]. In metazoans, the apoptosis pathway was first described over 35 years ago [3], and is now recognised as essential for normal growth and development, as well helping to guard against infections and the onset of cancer [1,4]. The process of apoptosis is initiated by the activation of death receptors, or by intracellular stress conditions [5]. This leads to a series of genetically controlled and ordered biochemical changes, resulting in morphological changes to the cell [5]. These include the condensing of chromatin, DNA breakdown, membrane changes, shrinkage of the cell and finally the formation of apoptotic bodies [6]. The membrane changes involved in apoptosis act as a signal for apoptotic bodies to be taken up by macrophages, preventing inflammation, as well as passing on information to scavenger cells on the cause of death [7]. In mammals, the process of apoptosis is rapid, removing cells within hours of initiation without evoking the inflammatory arm of the immune system.

Traditionally, apoptosis was thought of as a cellular activity exclusively relevant to multicellular organisms, but this view has recently been challenged. Morphological changes during cell death that are consistent with programmed cell death (PCD) have been reported for a range of unicellular organisms, including protozoan parasites [8-12]. The number of studies revealing PCD markers in unicellular organisms is rapidly increasing, and range across bacteria [13], slime moulds [14], yeast [15,16], algae [17], Trypanosomes [18-21], *Leishmania *[18,22], and *Plasmodium *[23-26]. The occurrence of PCD in unicellular parasites has proved controversial because, whilst the morphologies observed are consistent with apoptosis, it appears that the pathways involved are different to those in mammalian cells where the majority of research has focussed [8,27].

It is likely that like with other eukaryote cells [28] various forms of programmed cell death may be important in protozoan parasites including autophagy [29]. The detection of, and semantics for, parasite apoptosis is the focus of other papers within this thematic issue (Jiménez-Ruiz *et al.*, 'Apoptotic markers in protozoan parasites'; Picot *et al. *'Are protozoan metacaspases potential parasite killers?'). Here, we use the term 'apoptosis' to describe cells that have made the decision to die (analogous to suicide) as a strategy to improve transmission of surviving parasites. The distinction between apoptosis a strategy employed by parasites to die and apoptosis as simply the way in which parasites die when they are killed by host/vector factors is key; the former predicts apoptosis benefits all parasites in an infection, the latter predicts that a reduction in numbers is detrimental. As we are primarily interested in the evolutionary explanations for apoptosis to occur our focus is on apoptosis as a parasite strategy.

The occurrence of apoptosis in unicellular parasites is a challenge to explain because "Darwinian survival of the fittest" assumes organisms have evolved strategies to maximise their proliferation not their death. Here, we outline possible evolutionary explanations for apoptosis in protozoan parasites and suggest how they should be tested, with an emphasis on the importance of considering parasite ecology. We focus on malaria (*Plasmodium*) parasites as the application of an evolutionary framework to understand parasite life-history traits is better developed for malaria than other protozoan parasite species [30-33]. However, natural selection finds similar solutions to shared problems; therefore, it is likely that our message will be applicable more broadly to protozoan parasites. We start by outlining what is currently known about apoptosis in malaria parasites and the possible evolutionary explanations for why parasites would employ this strategy. We then go on to highlight the ecological factors which should be considered in choosing markers and conducting experiments on protozoan apoptosis, before suggesting possible future directions for testing the evolutionary explanations.

### Apoptosis in malaria parasites

**Figure 1:**
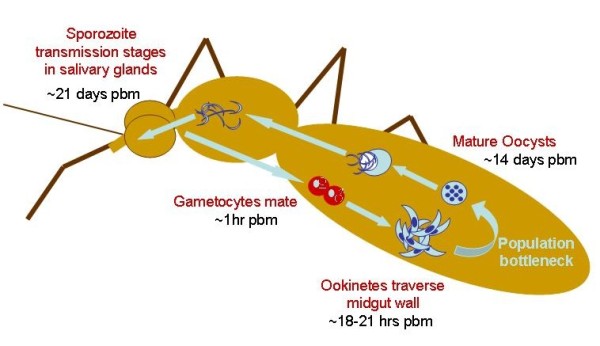
**Life cycle of malaria parasites within the mosquito vector (sporogeny)**. Timings show approximate estimates of the stages post blood meal (pbm) for the progression of *P. berghei *though *Anopheles stephensi*. Apoptosis has been observed in three species of malaria parasites at the ookinete stage. The ookinete to oocyst transition is a well known bottleneck in the parasite life-cycle. Estimated reductions in numbers at this point in the life cycle are variable but for *P.berghei *in *A. stephensi *one estimate is a 35 to 120 fold reduction in parasite numbers depending on the density within the vector [37].

Whilst in their vertebrate host's circulation, *Plasmodium *parasites produce asexual stage parasites, which go through rounds of replication within their host's red blood cells, and so maintain the infection. They also produce sexually differentiated transmission stages (gametocytes) which no longer replicate but if taken up by the mosquito vector provide the potential for transmission [34,35]. When taken up in a vector's blood meal, gametocytes must immediately differentiate into male and female gametes and mate (Figure 1). Within 18-20 hours post fertilisation, each zygote transforms into a motile ookinete, which traverses the midgut wall and invades the epithelium of their vector. Here, each ookinete differentiates into an oocyst and divides asexually to produce thousands of sporozoites. When an oocyst ruptures, its sporozoites are released into the haemocoel to migrate to the salivary glands, ready to be injected into new hosts [34,36]. This whole process, termed sporogony, takes around 21 days for *P. berghei *in *Anopheles stephensi *[37].

**Figure 2:**
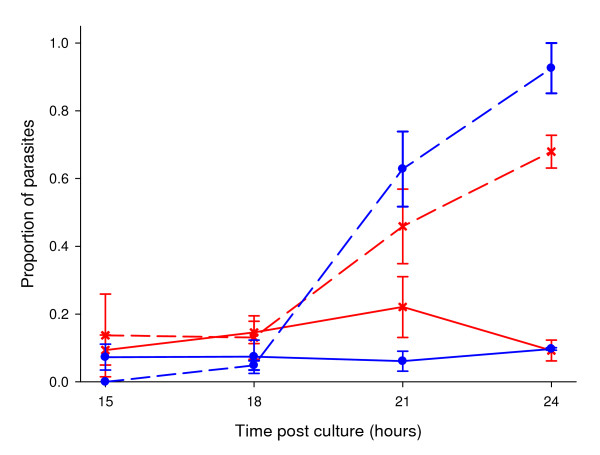
**Markers for apoptosis vary over time and between species**. Graphs show the proportion of ookinete stage parasites displaying DNA fragmentation as measured by TUNEL (solid lines) and Caspase-like activity (dashed lines) as measured by CaspaTag™ Pan-Caspase *In Situ *Assay Kit, Fluorescein in conjunction with propidium iodide (Chemicon international, USA) in *Plasmodium berghei *(red lines with crosses) and *Plasmodium yoelii *(blue lines with circles). Bars show standard errors of the mean and values are based on between 3 and 6 individual infections per time point. For each mouse 8 ookinete cultures were set up which were incubated for 14 hours before the cultures from each mouse were pooled and purified for ookinetes using macs ls cell separation columns (Miltenyi biotec). The resulting purified ookinetes were then alliquated into 8 individual 1 ml cultures (1 per time point for 2 assays) containing complete ookinete media. These cultures were then returned to the incubator until the time relevant time point (15, 18, 21 or 24 hours post culture set up). More detailed methodology is available in Additional file 1.

Recent research has revealed that large numbers of ookinete stage parasites display a variety of apoptosis markers (e.g. condensed chromatin, fragmented DNA, caspase-like activity, translocation of phosphatidylserine and loss of mitochondrial membrane potential) (Table 1). This includes observations in the rodent malaria parasite *P. berghei *[24], as well as the human malaria parasite *P. falciparum *[25]. There is also evidence of apoptosis markers in zygotes of *P. berghei *[24] and in asexual stages of *P. falciparum *after treatment with the anti-malarial drug chloroquine [23,26,38] and a common apoptosis inducer staurosporine [38]. Our own data provide further evidence for apoptosis in the ookinete stage of *P. berghei *and also the first evidence for *P. yoelii *ookinetes (Table 1; Figure 2; Additional file 1). Furthermore, previously published accounts of *Plasmodium *parasites displaying 'crisis' or 'degenerate' forms may provide earlier examples of PCD in malaria [39-43]. Controlled experimental approaches have demonstrated that this phenomenon occurs independently of mosquito and host immune cells and is not unique to *Plasmodium *parasites; evidence for apoptosis across a range of protozoan parasites (including *Leishmania, Trypanosoma *and *Toxoplasma *spp.) is rapidly accumulating [18,19,22,44].

**Table 1:** Variation in rates of apoptosis and temporal patterns observed in malaria parasites.

Species	Life cycle stage	ref	condition	Marker	Detection method	Proportion positive
*P. berghei*	ookinetes		*In vitro*	Condensed chromatin	Acridine orange (Sigma)	18 hrs - 15.5 (±1.06)%
						**18 hrs - 34.5 (±1.76)%**
		[83]	in PBS suspension			**22 hrs - 55.8 (±13.68)%**
						**26 hrs - 49.01 (±5.51)%**
				
				Fragmented DNA	TUNEL (histochemical, Calbiochem, UK)	**18 hrs - 48.55 (±6.01)%**
			or			**22 hrs - 64.19 (±6.09)%**
						**26 hrs - 69.89 (±2.81)%**
				
			**In RPMI**	Caspase-like activity	CaspaTag (Chemicon international, USA)	18 hrs - 17.0 (±2.12)
						**18 hrs - 30.15 (±2.14)%**
						**22 hrs - 43.8 (±1.53)%**
						**18 hrs - 47.72(±3.93)%**
				
				Translocation of phosphatidylserine	Annexin V- FITC apoptosis detection kit (Sigma, UK)	**18 hrs - 19.57 (±1.88)%**
						**22 hrs - 28.33 (±5.61)%**
						**26 hrs - 30.12 (±2.75)%**
				
				Mitochondrial membrane potential	JC-1 assay kit (Molecular Probes, UK)	18 hrs - 34.38 (±2.95)%
	
	ookinetes	[24]	*In vitro ***In RPMI**	Condensed chromatin	Acridine orange (Sigma)	**24 hrs - 31%**
						**36 hrs - 80%**
			
	ookinetes & zygotes mix		*In vivo*	Condensed chromatin	Acridine orange (Sigma)	18, 20 & 24 hrs - all over 60%
	
	ookinetes	[77]	*In vitro*	Translocation of phosphatidylserine	Annexin-FITC Apoptosis Detection Kit (Sigma, UK)	<3% (assay time not reported)
				
				Fragmented DNA	ApopTag^® ^Fluorescein In Situ Apoptosis Detection Kit (Chemicon International)	No positive cells observed (assay time not reported)
				
				Condensed chromatin	Acridine orange (Sigma)	No positive cells observed (assay time not reported)
				
				Caspase-like activity	CaspaTag (Chemicon international, USA)	**21 hrs - 3.8 (±0.05)%**
						**24 hrs - 14 (±9.00)%**
	
	ookinetes	*	*In vitro ***In RPMI**	Caspase-like activity	CaspaTag (Chemicon international, USA)	**15 hrs - 13.70 (±12.20)%**
						**18 hrs - 13.06 (±6.42)%**
						**21 hrs - 45.90 (±11.00)%**
						**24 hrs - 67.94 (±4.83)%**
			
	ookinetes		*In vitro ***In RPMI**	Fragmented DNA	*In situ *cell death detection kit, Flourescein (Roche)	**15 hrs - 9.38 (±4.44)%**
						**18 hrs - 14.57 (±3.29)%**
						**21 hrs - 22.08(±8.96)%**
						**24 hrs - 9.24 (±3.09)%**
	
	ookinetes	$	*In vitro ***In RPMI**	Caspase-like activity	CaspaTag (Chemicon international, USA)	**18 hrs - 20.06 (±3.50)%**

*P. yoellii*	ookinetes	*	*In vitro ***In RPMI**	Caspase-like activity	CaspaTag (Chemicon international, USA)	**15 hrs - No positive cells observed**
						**18 hrs - 4.85 (±1.40)%**
						**21 hrs - 62.8 (±11.10)%**
						**24 hrs - 92.59 (±7.41)%**
			
	ookinetes		*In vitro ***In RPMI**	Fragmented DNA	*In situ *cell death detection kit, Flourescein (Roche)	**15 hrs - 7.29 (±3.84)%**
						**18 hrs - 7.41 (±4.90)%**
						**21 hrs - 6.09 (±2.92)%**
						**24 hrs - 9.70 (±0.36)%**

*P. falciparum*	ookinete	[25]	*In vivo*	Fragmented DNA	TUNEL (histochemical, Calbiochem, UK)	24 hrs - 67.8 (±2.82)%
	
	Asexual blood stages (trophozoites & schizonts)	[23]	*In vivo *after treatment with chloroquine	Loss of mitochondrial transmembrane potential	Carbocyanine dye JC-1	Timings and proportions positive not reported
					
				Fragmented DNA	TUNEL (fluorescent, Roche)	
		
		[26]	*In vivo *after treatment with chloroquine	DNA laddering	After electrophoresis, Southern blotting and autoradiography, a ladder pattern observed	Timings and proportions positive not reported

*P. falciparum*	Asexual blood stages	[38]	*In vivo *after treatment with chloroquine (CQ) or staurosporine (ST)	Loss of mitochondrial transmembrane potential	Cell-permeable lipophillic cation probe JC-1 (Molecular probes, Eugene, USA)	10% in untreated cultures increased to 31% (CQ) and 25% (ST)
				Caspase-like activity	CaspaTag (Chemicon international, USA)	10% in untreated cultures increased to 34% (CQ) and 32% (ST)
				
				Fragmented DNA	ApoDirect DNA fragmentation assay kit (Clontech, San Diego, USA)	10% in untreated cultures increased to 27% (CQ) and 56% (ST)

Results organised by life-cycle stage and study. Although the majority of studies summarised here have focused on the ookinete parasite stage, there is still considerable variation in the proportion of cells judged to be apoptotic. This variation may be due to differences in experimental set up between labs, e.g. the nutrients available to parasites and the densities of cultures. Assays of apoptosis may also vary at specific time points in the proportions of positive cells depending on the time scale of the processes being assayed. When parasites are assayed over a time course there is a general trend for an increase in positive cells with time. * Pollitt *et al. *reported here (figure 2) $ Pollitt *et al. *reported here (figures 4 & 5)

Protozoan parasites cause some of the most serious infectious diseases of humans, livestock, wildlife, and companion animals. The discovery that these unicellular organisms undergo apoptosis, and that the underlying molecular and cellular processes appear to differ from those of multicellular eukaryotes, offers a new paradigm for medical and veterinary interventions [23]. However, these differences have also resulted in controversy over which methods, terminology, and markers are appropriate for protozoan parasites. This debate must be resolved before parasite apoptosis can be understood: from the proximate genetic, molecular and cellular mechanisms that orchestrate cell death, to the ultimate evolutionary explanations for the existence of PCD in unicellular organisms.

### Evolutionary explanations for apoptosis

For unicellular parasites, suicide may appear to be a counter-intuitive strategy when organisms are expected to have evolved to maximise their proliferation. However, uncontrolled replication often is not the best strategy for parasites as this may lead to the host or vector dying before there is a chance for transmission [45]. Evolutionary theory therefore suggests that under certain conditions parasites will be selected to display prudence in order to prevent premature death of their host, maximising the time and resources available for transmission and therefore their fitness [45-47]. Apoptosis in single celled organisms can be viewed as an extreme form of prudence and a cooperative (helping) behaviour.

It has been suggested that protozoan parasites may undergo apoptosis as a cooperative behaviour in order to prevent killing the host/vector [20,21,24,48,49]. However, evolutionary theory predicts that a cooperative behaviour will only evolve if the cost to the individual performing the behaviour (the actor) is outweighed by the benefit to the recipients weighted by the relatedness of the actor to the recipients [50,51]. A parasite committing suicide (via apoptosis) is an extreme form of cooperation, and obviously death is the highest cost payable. This means that the relatedness between apoptosing parasites and the survivors must be high, and that there must be a substantial benefit provided to the survivors, otherwise a trait as costly as suicide could not have evolved. If infections are initiated by one or very few clones then relatedness will be high. In this case, the question becomes 'how can overall transmission success (fitness) be improved by a reduction in parasite density?' For malaria parasites, there are at least two non-exclusive reasons why lowering parasite density could increase the chance of successfully completing the life cycle in the vector and being transmitted.

First, capping the density of ookinetes within a mosquito may prevent premature mortality of the vector by limiting damage caused either by ookinetes traversing the midgut epithelium or by later stages in sporogony [52]. Limiting damage to mosquitoes may be particularly important for malaria parasites as the development time required to be transmissible from the salivary glands (~3 weeks) is long compared to the average life expectancy of mosquitoes in the wild, which some estimates put at as low as 1-2 weeks for adult females of the *Anopheles *species [53]. Therefore, slight variations in the mortality rates of mosquitoes could have a significant effect on parasite transmission. The effect of malaria infection on mosquitoes is controversial with some studies finding a positive correlation between oocyst density and mosquito mortality, but others finding no evidence of a cost to lifespan [54]. These contrasting results may be due to artificially good conditions in lab experiments masking negative effects of malaria infection [54]. However, if parasites employ apoptosis to limit damage to mosquitoes then a benign effect of infection should not be surprising. Second, ookinetes at high density could directly effect sporozoite production if oocysts at high density compete for access to limited resources (e.g. nutrients), or indirectly by inducing stronger mosquito immune responses. Little is known about the developmental requirements of oocysts, but malaria infected mosquitoes are more likely to sugar-feed and divert resources away from reproduction (through apoptosis of their ovary cells) [55,56], suggesting that malaria infection causes a significant energetic burden.

Cooperation is widespread and recent advances in mathematical theory and empirical methods have revealed that the same general principles explain the evolution of cooperation and conflict across a wealth of taxa (from bacteria to insects to humans). This framework predicts that ookinetes will undergo apoptosis when closely related parasites benefit, and ookinete numbers are high enough to negatively affect mosquito lifespan or sporozoite production. These predictions provide the specific, testable, hypotheses that: the proportion of apoptosing ookinetes will: (i) be density dependent and increase with the number of ookinetes in the midgut; and (ii) be greatest when ookinetes in an infection are clonally related and decrease as the genetic diversity of parasites sharing a vector increases. Examples of unicellular parasites cooperating with relatives in a density-dependent way are common [57,58]. Many of the best examples of this come from bacteria that form complex structures called biofilms to provide protection from the host immune response or antibiotic drugs [59], and bacteria that forage cooperatively to extract iron in a usable form from their host [60].

Despite the generality of the evolutionary principles that explain cooperation, the suggestion that apoptosis in malaria parasites is a social trait is controversial. Whilst in bacteria, quorum sensing mechanisms have been described to explain the coordination of behaviour [61], as yet, no specific quorum signalling system has been found for malaria parasites. However, evidence that malaria parasites respond to changes in their within-host environment by altering their resource allocation decisions show that they can detect and respond to factors such as the presence of competitors and variation in resource availability [31,62,63]. The predictions for why parasites undergo suicide are clear and testing them will resolve whether parasite apoptosis has been shaped by natural selection to enable parasites to cooperate with their kin. As with all emerging and interdisciplinary fields, undertaking the key, conceptually simple experiments required to test these predictions is constrained by the limitations of the methods and techniques available. For example in bacteria targeted disruptions have been useful in testing the fitness consequences of specific phenotypes (e.g. [64]), however, specific candidate genes for apoptosis in malaria are lacking and complex traits are difficult to disrupt. In the next section we outline the methodological constraints that currently impede the collection of data of high enough quality to undertake quantitative tests of the evolutionary explanations for parasite apoptosis. Given the medical and economic implications of malaria parasites and the drive to develop transmission-blocking intervention strategies, understanding their transmission biology from an evolutionary perspective is also timely and important.

### Ecological considerations: applying assays for morphological markers

We suggest that to examine the evolutionary causes and consequences of apoptosis in malaria parasites, their ecology must be taken into account when deciding which assays to use and how best to apply them. In Table 2 we outline the markers and assays available, highlighting their suitability (from an ecological perspective) for use with malaria parasites, and we discuss the general issues below.

**Table 2:** Summary of some commonly used markers for apoptosis.

Marker of apoptosis	Example of assay used	Method of detection	Practical considerations	Relevance for malaria ecology
Activation of caspase-like molecules	CaspaTag (Chemicon international, USA)	A fluorescent labelled general caspase inhibitor (FAM.VAD.fmk (green) and SR.DEVD.fmk (red)) binds to active caspase within the cell. Positive cells fluoresce under fluorescent microscope	- Quick and easy to use	The role of caspase-like molecules is controversial in protozoan parasites, therefore it is not possible to be certain that apoptosis is being detected. However, if caspase molecules are a reliable marker they would be useful as an early marker of induction.
			-Results not as clear as with TUNEL	
			- Viability tests can be performed in conjunction	
			- Large scale experiments possible	
			The caspase inhibitor used in the caspase assay is broad spectrum and therefore may cross-react with unrelated molecules.	

Depolarisation of mitochondria outer membrane	JC-1 assay kit (Molecular Probes, UK)	JC-1 is a cationic carbocyanine dye that accumulates in mitochondria. Loss of mitochondrial membrane potential can be detected by the shifting of emission of fluorescence from orange (polarised mitochondrial membrane) to green (depolarised mitochondrial membrane).	- Quick and easy to use	The role of mitochondria in malaria apoptosis not well established. However, if markers prove to be reliable they would be useful as an early marker of induction.
			- Viability tests can be performed in conjunction	

Condensed chromatin	Acridine orange (Sigma)	Differentially stains SS and DS nucleic acids - enables the detection of condensed chromatin. Apoptotic cells should show an intense red staining in nucleus	- Quick and easy to use	Good relevance as we would expect this process to be the same for mammalian and protozoan cells.
			False positives possible and Results not as clear as with TUNEL	
			- Viability tests can be performed in conjunction	
			- Large scale experiments possible	

Translocation of phosphatidylserine to outer cell membrane	Annexin V- FITC apoptosis detection kit (Sigma, UK)	Positive display green annexin labelling on the cell surface, which can be detected by fluorescent microscopy	- Quick and easy to use	May not be relevant for malaria cells for two reasons.
			- Results not as clear as with TUNEL	1. The cell membrane of protozoan parasites is very different to that of mammalian cells.
			- Viability tests can be performed in conjunction	2. The ultimate reason for mammalian cells expressing phosphatidylserine on the outside of apoptotic bodies in order to be taken up by phagocytes, is not relevant for the mosquito midgut.
			- Large scale experiments possible	

Fragmented DNA leading to the generation of fragments with 3'OH groups	*In situ *cell death detection kit, Flourescein (Roche)	DNA of fixed and permeabilized cells labelled by the addition of flourescein dUTP at strand breaks by terminal transferase. Flourescein then detected by fluorescent microscopy (figure 3)	- More laborious than using Acridine orange, CaspaTag or Annexin V detection	Good relevance as we would expect this process to be the same for mammalian and protozoan cells. However as DNA fragmentation is thought to be a late process in apoptosis may only see markers at a later time point than induction of apoptosis pathways.
			- Gives clear unambiguous results.	
			- Requires cells to be dead so cannot perform viability tests in conjunction.	
			- Slides can be stored (at 5°C) for a few days allowing later analysis and therefore large scale experiments.	Some necrotic cells may show positive.

Morphological Markers e.g. membrane blebbing and formation of apoptotic bodies	Electron microscopy	Observation of cell morphology under electron microscope to detect membrane blebing or formation of apoptotic bodies	Time consuming and expensive	Malaria parasite cells differ in structural aspects from mammalian cells, it is therefore not clear whether the structural changes observed in mammalian cells would be relevant for these parasites. The ultimate reasons for formation of apoptotic bodies to be taken up by macrophages also not relevant in the mosquito midgut.
			- Not a good basis for morphological changes seen in malaria apoptosis	
			- Requires cells to be dead so cannot perform viability tests in conjunction.	
			- Large scale experiments not possible but may be useful in conjunction with other assays	

**Detecting cell viability**				
Propidium iodide (PI)	Propidium iodide (Roche)	Stain is taken up in cells with compromised membranes causing cells to display a red fluorescence.	- Quick and easy to use	Useful method for assessing viability of cells which can be used in conjunction with other assays of apoptosis.
			- Cells must be viewed quickly after application	
			- Can be used in conjunction with assays on live cells e.g. CaspaTag.	

Particular reference is paid to their ecological and practical considerations when using to test evolutionary predictions. For more details on the markers of apoptosis in protozoan parasites see paper by Jiménez-Ruiz *et al. *('Apoptotic markers in protozoan parasites') in this thematic.

#### Which markers matter?

Many kits for assaying markers of apoptosis in mammalian cells are commercially available. However, it is not clear which markers are most suitable for measuring apoptosis in protozoan parasites. For example, one often used marker for apoptosis in mammalian cells is the translocation of phosphatidylserine to the outside of the cell membrane, which is detected using an annexin assay. In mammals phosphatidylserine provides a signal for the apoptotic cell to be engulfed by phagocyctes, which prevents the cell from disintegrating and the resultant debris from causing inflammation. Whilst such a 'tidy death' is clearly advantageous in a muticellular organism, it may not be an applicable concern for single-celled ookinetes in the mosquito midgut. However, the presence of this marker in yeast and leishmania suggests that it may have additional functions [16,65]. In leishmania it is thought that phosphatidylserine translocation acts as a form of 'apoptotic mimicry' which aids the parasite in infecting macrophages [65]. In yeast the reason for the membrane altering during apoptosis is not yet known, however an interesting possibility is that it could act as a signal to other yeast cells.

Another common marker for apoptosis is caspase activity. In mammalian cells, classical apoptosis is triggered by the activation of caspases, which are apoptosis-specific cysteine proteases within the clan CD [66]. Part of the controversy surrounding the characterisation of parasite apoptosis stems from the absence of 'true' (canonical) caspases in protozoans [27,67]. However, ancient caspase homologues (known as metacaspases) are present in the genomes of plants, fungi and protozoa [68]. In plants and fungi metacaspases have been shown to be involved in apoptosis [69] and four metacaspases have been identified in the *Schistosoma mansoni *and *S. japonicum *[67,70] which could also play a role in a form of PCD. In *Plasmodium *there are three metacaspases (PxMC1, PxMC2 and PxMC3), and like the mammalian counterparts, they all posses a defined pro-region and a catalytic domain that is indicative of the clan CD, family C14 caspases. It has been suggested that one or more of these metacaspases can carry out a functionally analogous biological role to metazoan caspases, and although data are scarce, they have been linked to programmed cell death in some unicellular organisms [44,71-73]. Of the three metacaspases in *Plasmodium*, MC1 may represent the best candidate enzyme to be involved in a form of PCD in *Plasmodium *as it is the only MC that has the required predicted catalytic cysteine and histidine residues in the correct context for an active enzyme, and is typified by the *P. falciparum *enzyme PfMC1 (PF13_0289) ([74], Sajid personal communication) However, it is now evident, from work on mammalian cells, that beside the caspase dependent form of apoptosis a caspase independent form can also occur [8,75].

The role of metacaspases in parasite apoptosis is controversial, and contrasting conclusions have been drawn from different experiments [8,27,76]. Supporting data come from studies showing that the addition of the broad spectrum caspase inhibitor Z-VAD.fmk results in a reduction of the number of ookinetes displaying a variety of apoptotic morphologies, and also a doubling in the number of oocysts in the mosquito midgut [24]. In contrast, one study examining a *P. berghei *line in which PbMC1 has been deleted, found no loss of apoptosis (judged using CaspaTag) and therefore concluded that it may be a functionally redundant gene [77]. However, these authors did not apply any other markers to their knock out line and found very low rates of phosphatidylserine translocation and no DNA fragmentation or chromatin condensation with their wild-type line. Our own research has shown that when compared to genetically intact parasites, this PbMC1 knock out line reaches higher ookinete and oocyst densities in infected mosquitoes and that this translates into lower sporozoite production and higher mosquito mortality (Pollitt *et al*., unpublished data). This demonstrates there is a cost to sporogony at high parasite densities and suggests that PbMC1 mediates ookinete numbers although it is not yet clear if this is through apoptosis.

Recent research has implicated clan CA cysteine proteases in chloroquine mediated apoptosis [38]. However, the use of inhibitor/probes developed for use in humans or other systems with canonical caspases should be viewed with caution. To date there are no proteases from Plasmodium that have a specificity for Asp at P1 (as per Val-Ala-Asp (P3-P2-P1)) of the caspase selective probes. This together with irreversible nature (fmk) of these probes is likely to lead to off target inhibition and the clan CA may be amongst these off target hits [78]. This problem is compounded if these inhibitors are used either at very high concentrations or they are used over an extended time period.

#### How should assays be applied?

One of the attractions of using the activation of caspase-like molecules as a marker for apoptosis is the ability to assay cells in the early stages of apoptosis. This is important for studies aiming to assay apoptosis at biologically relevant time points. For example, ookinetes begin to develop from retort (immature) forms at around 8 hours post fertilisation and begin to invade the gut epithelium at 18-20 hours for *P. yoelii*, although these timings vary between malaria parasite species [36]. If some ookinetes undergo apoptosis in order to provide a benefit to others, we expect apoptosis will be initiated before 18-20 hours post fertilisation. Therefore, it is important to assay apoptosis rates before ookinetes have either died or transformed to the next life cycle stage. Assays for depolarisation of mitochondrial membrane potential may also have the advantage of being an early marker of apoptosis [79,80], however, as with many of assays discussed here the relevance for malaria parasite cells has not yet been established. Intuitively, assaying the activation of death executors would appear to be the best approach when testing whether levels of apoptosis are linked to developmental schedule, but early apoptotic mammalian cells can be saved [81] and it is not yet known at what stage parasites become irreversibly committed to dying.

**Figure 3:**
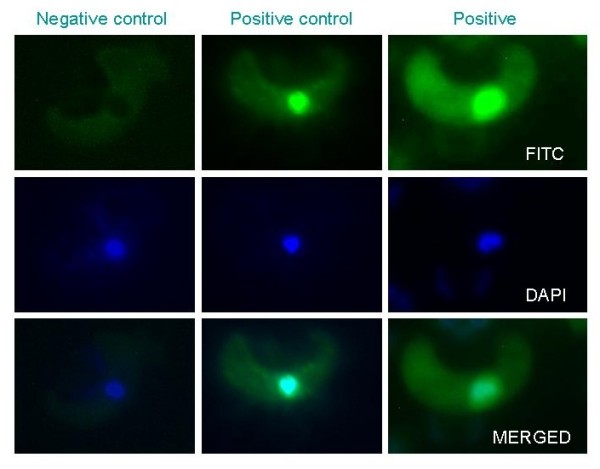
**Identification of DNA fragmentation by fluorescent TUNEL assay**. Images of *P. berghei *ookinetes with fragmented DNA marked using a TUNEL assay (*In situ *cell death detection kit, Fluorescein, Roche) at 21 hours post culture. Positive cells show a bright green nucleus. Negative controls were incubated with only the label solution without the enzyme and positive controls had DNA strand breaks induced with DNase 1 recombinant (3 units/ml for 10 mins at 20°C) before labelling (DNase 1 recombinant, grade 1, Roche). DAPI staining was used to check the location of the nucleus. More detailed methodology is available in Additional file 1.

A possible solution to this problem is to assay morphologies observed at the end of the apoptosis program, such as DNA fragmentation. However, this approach may complicate the ambition of examining apoptosis in a biologically relevant timeframe. The key question is how long does the apoptosis program in malaria parasites take? For example, if ookinetes are predicted to initiate apoptosis at 18 hours post fertilisation, how much later should DNA fragmentation be assayed? We show a preliminary examination of this issue in Figure 3. The proportion of cells with fragmented DNA at the time points measured are significantly lower than those displaying caspase-like activity, but clearly more studies are required to characterise the time lags between the activation of apoptosis and the appearance of the resulting morphologies. These studies should be designed with reference to the developmental schedules of parasite species that undergo sporogony at different temperatures. Another problem with assaying late-stage apoptosis morphologies is the validity of combining assays to distinguish between apoptotic cells and necrotic cells. Furthermore, any temporal variation in how individual parasites initiate and progress through apoptosis will make it difficult to distinguish between cells in a sample that are healthy, undergoing apoptosis, have died by apoptosis, or have died by necrosis. This could be further complicated by the fact that assays such as TUNEL for DNA fragmentation can also be positive in necrotic cells. This all points to the importance of having reliable time-lines for the changes associated with the different forms of cell death.

In our experience, measuring DNA fragmentation by fluorescent TUNEL assay appears to provide repeatable and non-subjective results (positive cells show an obviously fluorescing nucleus; Figure 3). Fragmentation of DNA is also a well defined end point to a program of programmed cell death. However, because TUNEL assays are applied to fixed and permeabilized cells, parasites that do not display DNA fragmentation cannot be further characterised as healthy or necrotic. For this reason, assays that can be applied to live parasites are very useful, such as CaspaTag (but see 'which markers matter' section). Because most apoptosis assays have been developed for mammalian cells, the protocols involved may not always be appropriate for live parasites. For example, ookinete stage malaria parasites develop at considerably lower temperatures and would experience significant stress and heat shock if treated at 37°C during assaying. Electron microscopy on parasite nuclei can be useful to verify and compare processes in order to reliably discriminate between apoptosis and other forms of programmed cell death. However, this is not practical for hypothesis testing on large numbers of cells. For most experiments wanting to study ecological variation then it is also necessary that assays allow high through-put of samples. In these situations assays on dead and fixed parasites that can be stored for later analysis (e.g. fluorescent TUNEL) are more practical.

In addition to studies that characterise parasite apoptosis programs, technical developments are required so that assays can be applied to large numbers of cells and enable their morphologies to be efficiently and accurately quantified.

#### Variation: noise or not?

A major challenge for evolutionary biology is explaining variation in traits observed across genotypes, and within the same genotype in different environments. The relative proportions of parasites recorded as undergoing apoptosis varies over time, between markers and across studies (see Table 1). This variation is common in evolutionary studies of phenotypic traits and initially seems difficult to interpret. The challenge is to identify patterns and understand what is driving them. Recent studies have revealed that malaria parasites detect and respond to subtle changes in the conditions they encounter during infections by altering traits such as investment in gametocytes and their sex ratio [31,62,63,82]. These conditions include the density of clone-mates and genetic diversity of co-infecting parasites, which are also the factors predicted to influence levels of apoptosis. Therefore, variation across studies in infections and/or experimental set up may result in differences in the cues that parasites experience or their ability to detect this information.

**Figure 4:**
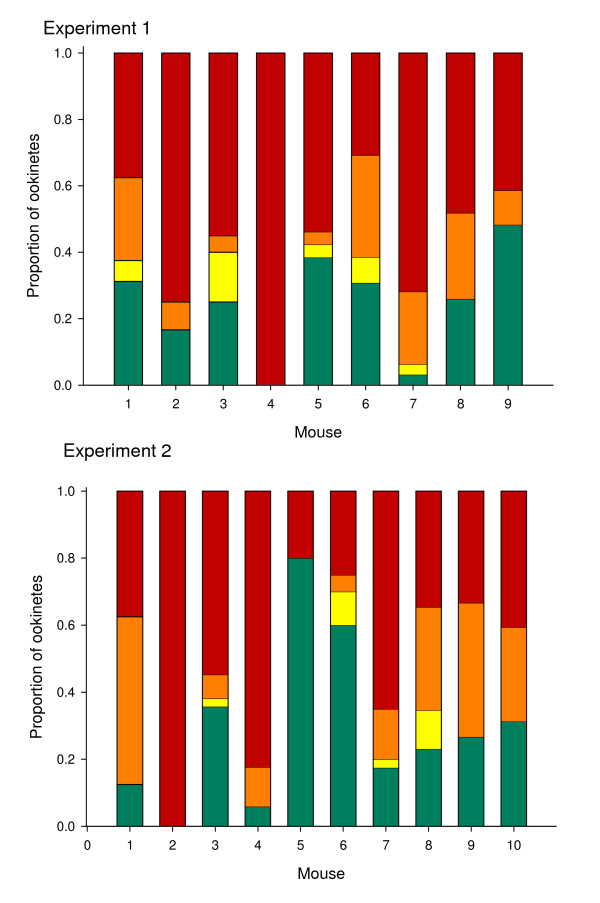
**Replicate experimental infections show variation for proportions of parasites showing caspase-like activity and viability**. Two sets of replicate experiments were set up by infecting 10 male MF1 mice 8-10 weeks old per replicate with 10^7 ^*P. berghei *parasites after pre-treatment with Phenylhydrazine 2 days pre-infection (120 mg/kg). One mouse from experiment 1 failed to become infected so was removed from the study. Cultures were then set up 4 days post infection. After 18 hours ookinetes were purified using MACS LS cell separation columns (Miltenyi biotec) and a minimum of 30 parasites were assayed for caspase like activity and viability using CaspaTag™ Pan-Caspase *In Situ *Assay Kit, Fluorescein in conjunction with propidium iodide (Chemicon international, USA). Green indicates healthy ookinetes negative for caspase-like activity with intact membranes, yellow indicates early apoptotic ookinetes displaying caspase-like activity with intact membranes, orange indicates late apoptotic ookinetes displaying caspase-like activity but also compromised membranes and red indicates dead cells with compromised membranes [25]. More detailed methodology is available in Additional file 1.

**Figure 5:**
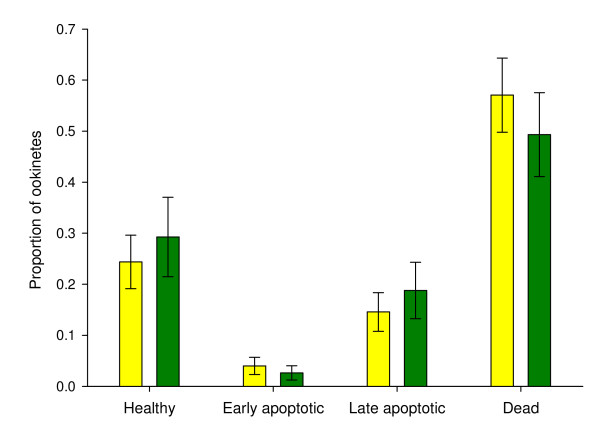
**Between experiments markers of apoptosis and death are repeatable**. Data taken from the experiments described in figure 4. Average proportion of ookinetes classified into 4 categories (healthy, early apoptotic, late apoptotic or dead) across 9 replicate infections for experiment 1 (yellow) and 10 replicate infections for experiment 2 (green). Although there is variation for replicate infections within experiments (figure 4) there is no significant variation between the cells categorised into each condition between 2 experiments carried out on different days (χ^2 ^= 5.81 (3 df), p > 0.1). Error bars show the standard error of the mean.

Our data show that even within controlled replicate infections - initiated with the same infective dose of the same parasite clone in the same batch of hosts - there is variation in the proportion of parasites displaying markers of apoptosis (Figure 4). This may be due to parasites responding to subtle variation in parasite densities or other aspects of their within-host environment, such as immune challenge or anaemia. However, when the data from individual infections within an experimental group are combined and we compare two replicate experimental groups, we see that individual replicates are noisy but patterns are consistent (Figure 5). This suggests that it is possible to reliably detect patterns but large sample sizes (number of independent infections) and standard conditions are required. Also, where possible, variables such as parasite density should be recorded in order to control for its potential influence on rates of apoptosis.

Parasite ecology may also be important for understanding variation in apoptosis behaviour between different parasite species. For example, a comparison of our data for rates of apoptosis observed in *P. berghei *and the related rodent malaria species *P. yoelii *(Figure 2) reveals significantly different patterns of caspase-like expression over time (species*time χ^2^_1 _= 4.38, p = 0.046). The proportion of parasites assaying positive with TUNEL was not significantly different over time (χ^2^_1 _= 0.001, p > 0.5). However the proportion positive at 21 hours was significantly lower in *P. yoelii *(t = 2.37, df = 8, p = 0.045). Given the debate over the reliability of using caspase-like activity as a marker of apoptosis in *Plasmodium*, we propose that a more accurate picture is gained from focussing on the TUNEL positive cells. Can differences in species ecology explain the difference in levels of DNA fragmentation in these species? A possible explanation is that as *P. berghei *parasites can reach considerably greater oocyst densities than *P. yoelii*, higher rates of apoptosis are required for *P. berghei *survivors to gain a benefit in terms of increased transmission success. With this in mind it would be interesting to examine the rates of apoptosis in other malaria parasite species.

## Conclusions

The discovery of apoptosis-like cell death in single celled protozoans such as malaria parasites provides an exciting challenge for evolutionary biology to explain and a new direction for intervention strategies. Progress in both of these fields requires evolutionary biologists to work together with cell biologists to develop reliable high throughput assays to study variation in apoptosis in response to the key parameters of parasite density and infection genetic diversity. At the same time, debates on the best markers for assaying apoptosis and appropriate terminology need to be resolved.

## Competing interests

The authors declare that they have no competing interests.

## Authors' contributions

LCP collected the results presented here and wrote the first draft of the manuscript, SER assisted in the design of experiments and helped to draft the manuscript. NC, SKM, MS all participated in the formation of the final version of the manuscript and helped to draft specific sections. All authors read and approved the final manuscript.

## Supplementary Material

Additional file 1 **Supplementary information**. More detailed Methods for our data presented here can be found in the supplementary information. Click here for file
