# Supplementary material for: Investigating the evolution of apoptosis in malaria parasites: the importance of ecology
Source: Parasit Vectors. 2010 Nov 16;3:105. doi: 10.1186/1756-3305-3-105 (PMC3136143; doi:10.1186/1756-3305-3-105)
Supplement: Additional file 1 — Supplementary information. More detailed Methods for our data presented here can be found in the supplementary information. [file 1756-3305-3-105-S1.DOC]

**Supplementary information**

**Materials and methods**

***Infections and culturing parasites***

We used the rodent malaria parasites *P. berghei* 820 and *P.yoelii* wild type from the WHO Registry of Standard Malaria Parasites (The University of Edinburgh). Infections were initiated in male MF1 mice (8-10 weeks old) which had been pre-treated with Phenylhydrazine (PHZ) (60mg/kg (3 days prior to infection) or 120mg/kg (4 days prior to infection) for *P.yoelii* and *P.berghei* infections respectively). This treatment stimulates the erythropoietic response in the mice encouraging the establishment of the malaria infection and the production of gametocytes [83]. Infections with both species were initiated with 10*7 parasites and infected blood was collected for culturing on day 3 or 4 post infection for *P.yoelii* and *P.berghei* respectively. Ookinete cultures were set up with 75μl of infected blood in 5mls of complete ookinete culture media (RPMI + 10% fetal calf serum, pH 8) and incubated at 24°C or 21°C respectively for *P.yoelii* and *P.berghei*. After incubation ookinete cultures were purified using MACS LS cell separation columns (Miltenyi biotec).

***Detection of Caspase-like activity and cell viability***

Caspase-like activity was measured using CaspaTag™ Pan-Caspase *In Situ* Assay Kit, Fluorescein iodide (Chemicon international, USA) following the manufacturers instructions except for modification of incubation temperature which were changed from 37°C to 24°C or 21°C respectively for *P.yoelii* and *P.berghei* cells. Previous studies have shown that lowering the incubation temperature to a suitable level for ookinete development does not alter the proportion of cells showing caspase-like activities and that higher temperatures result in increased death in ookinetes as measured by membrane viability [25]. CaspaTag utilises a carboxyfluorescein-labeled fluoromethyl ketone peptide inhibitor of caspase (FAM-VAD-FMK) which acts as a non-cytotoxic fluorescent broad-spectrum caspase inhibitor that binds covalently to active caspases in living cells. After incubation cells which contain the bound reagent (indicating caspase-like activity) will glow green when analysed by fluorescence microscopy.

***Fragmented DNA***

DNA fragmentation was measured using TUNEL *In situ* cell death detection kit, Fluorescein (Roche) following the manufacturers instructions. Purified ookinete cultures were centrifuged, smeared and fixed onto a glass slide with 4% Paraformaldehyde in PBS (pH 7.4, 1 hour incubation at 15-21°C). Cells were then permeabilised using 0.1% Triton X-100 in 0.1% sodium citrate (2 minutes on ice). Fixed permeabilised cells where then incubated with the TUNEL reaction mixture which labels DNA strand breaks, by terminal deoxynucleotidyl transferee (TdT), which catalyzes polymerization of labelled nucleotides to free 3’-OH DNA ends in a template-independent manner (TUNEL-reaction). Flourescien labels incorporated into the nucleotide polymers are can then detected by fluorescence microscopy (the nucleus of positive cells fluoresce bright green, see figure 3). After incubation with the tunnel reaction mixture VECTASHIELD mounting medium with DAPI (vector laboratories) was added to samples to prevent the fluorescence fading and allow the location of the nucleus to be confirmed.

***Cell viability***

Cell viability was measured using propidium iodide (PI) (250µM/ml) (Chemicon international, USA) which enters cell with permeabilised membranes resulting in red fluorescence.
